# Editorial: Machine learning algorithms for brain imaging: new frontiers in neurodiagnostics and treatment

**DOI:** 10.3389/fninf.2026.1794013

**Published:** 2026-02-09

**Authors:** Avinash Tandle, Shailesh Appukuttan, Hamed Honari

**Affiliations:** 1 Department of Electronics and Telecommunication Engineering, Mukesh Patel School of Technology Management and Engineering, Shri Vile Parle Kelavani Mandal's (SVKM's) Narsee Monjee Institute of Management Studies (NMIMS) University, Mumbai, India; 2 UMR7289 Institut de Neurosciences de la Timone (INT) Marseille, Marseille, France; 3 UMR7339 Centre de Résonance Magnétique Biologique et Médicale (CRMBM), Marseille, France; 4 Stanford University, Stanford, CA, United States

**Keywords:** deep learning, fMRI, machine learning, MRI, neurodiagnostics, neuroimaging

The field of neuroimaging has undergone profound transformation in recent years, driven primarily by rapid advances in machine learning (ML), and especially deep learning (DL), techniques. These computational innovations have been amplified by ongoing improvements in brain imaging modalities, such as structural MRI (sMRI), functional MRI (fMRI), diffusion tensor imaging (DTI), and positron emission tomography (PET), which now deliver increasingly high-resolution, multimodal views of brain structure, function, connectivity, and metabolism.

The intersection of neuroimaging and machine learning marks an inflection point in neuroscience; a confluence where vast, high-dimensional brain data meet computational power capable of turning complexity into clinically actionable insights. This convergence has led to the rapidly expanding domain of machine learning for neuroimaging, inviting a new generation of studies that leverage ML to transcend the limitations of traditional neuroimaging analysis.

Traditional statistical approaches and model-based techniques often struggle with the defining features of modern neuroimaging datasets: extreme high dimensionality, substantial heterogeneity across subjects/scanners/protocols, nonlinear patterns, and large-scale “big data” from consortia and multimodal fusion. In contrast, ML/DL paradigms provide powerful approaches to address the aforementioned challenges by virtue of their ability to learn complex, non-linear relationships in the underlying data through their architecture, end-to-end representation learning from raw or minimally processed data, capturing intricate spatiotemporal patterns that yield superior performance in tasks like disease classification, lesion segmentation, biomarker discovery, stratification and progrnosis, individualized treatment planning and predictive modeling.

Several challenges remain, such as overfitting, interpretability, explainability, computational demands, and generalization across diverse datasets. Nevertheless, this integration holds transformative potential for more accurate, individualized, and clinically translatable neuroscience insights.

This Research Topic was conceived to provide a platform for researchers involved in a variety of related domains such as neuroscience, medical imaging, and artificial intelligence to come together, and report their state-of-the-art findings in the application of ML to neuroimaging. Our objective was to address both the methodological and translational challenges involved, whilst also critically examining their clinical relevance, robustness, interpretability, explainability and ethical implications in this rapidly evolving field.

## Overview of contributions

The Research Topic comprises 13 peer-reviewed articles, including original research papers and methodological contributions, that collectively reflect the diversity and dynamism of current machine learning research in brain imaging. This Research Topic aims not only to push the methodological boundaries, but to showcase the contributions made to translate the ML-driven neurimaging into real-world neurodiagnostic and therapeutic applications.

In this Research Topic, several contributions focus on deep learning–based image analysis, proposing innovative architectures and training strategies for tasks such as brain segmentation, tissue classification, and feature extraction from high-dimensional neuroimaging data. These studies demonstrate how convolutional and representation-learning approaches can improve accuracy and robustness compared to conventional pipelines, while also reducing the need for manual intervention.

Another set of articles addresses predictive modeling and clinical decision support, leveraging machine learning to infer disease states, progression patterns, or treatment-relevant biomarkers from imaging data. These works illustrate the growing role of ML in moving beyond descriptive neuroimaging toward actionable neurodiagnostics and prognostication.

The Research Topic also includes studies emphasizing multimodal data integration, where complementary information from MRI, fMRI, PET, and related modalities is combined through advanced learning frameworks. Such approaches highlight the potential of machine learning to capture interactions across structural, functional, and metabolic dimensions of brain organization.

Importantly, several contributions engage with interpretability, explainability, generalizability, and validation, addressing key barriers to clinical translation. By incorporating explainable AI techniques, cross-dataset evaluations, and careful benchmarking, these studies underscore the necessity of trustworthy and reproducible machine learning models in neuroimaging research.

## Challenges, opportunities, and future directions

While the contributions in this Research Topic showcase the transformative potential of machine learning in brain imaging, they also highlight persistent challenges. These include limited dataset sizes, heterogeneity across datasets (such as scanner, site, protocols, unaccounted variability in the population), class imbalance, and the need for transparent decision-making (in particular advancement of interpretable and stable explainable techniques for black-box architectures) in clinical contexts. Addressing these issues will require continued methodological innovation, open data sharing, standardized evaluation protocols, and close collaboration between clinicians, neuroscientists, and machine learning experts.

Looking forward, the integration of machine learning with longitudinal imaging, multimodal data fusion, stringent benchmarking and embedding into real-world clinical workflows holds particular promise. Advances in explainable AI (XAI) and emerging causal modeling approaches may further bridge the gap between predictive performance and mechanistic understanding, ultimately supporting more personalized and effective neurotherapeutic interventions.

## Prospects—toward personalized neurodiagnostics and intervention

As we stand on the cusp of this new era, the trajectories emerge with profound potentials:

- Personalized diagnostics: ML-derived signatures may enable early detection of disease, even before overt symptoms, offering a critical window for timely intervention.
- Prognosis and treatment planning: ML models can stratify disease risk, predict progression trajectories, and tailor therapy decisions, especially as more multimodal datasets (e.g., neuroimaging combined with clinical, genomic, and biomarker data) become widely available.
- Real-world clinical deployment: With robust validation across centers and interpretable AI pipelines, ML-based neuroimaging tools may support radiologists and neurologists, augmenting but not replacing expert judgment.
- Discovery of novel biomarkers: Beyond known disease markers, unsupervised or self-supervised ML techniques might uncover latent imaging features, offering fresh insights into pathophysiology.

## Concluding remarks

Realizing this ambitious vision, however, requires more than algorithmic innovation alone. It demands large-scale multi-center collaborations to assemble diverse and representative datasets, standardization of acquisition and pre-processing pipelines for reproducibility. Rigorous external validation and benchmarking across independent cohorts and clinical settings are essential to establish generalizability. These efforts need to be accompanied by proactive ethical stewardship, including robust data privacy protections, bias mitigation (algorithmic and otherwise), fairness safeguards, and equitable access to ML-powered neurodiagnostics. In parallel, it is essential to prioritize scientific rigor, explainability, interpretability, and ethical responsibility in pursuit of higher accuracy and clinical trust. We are hopeful that the articles in this Research Topic will serve as a valuable resource for both researchers and clinicians alike, and that it may stimulate further interdisciplinary collaborations. The near future will witness much development at this intersection of machine learning, neuroimaging, and clinical neuroscience. The continued adaptation of ML approaches to neuroimaging holds us in good stead to push the boundaries of our understanding of brain function, and improve medical interventions for patients with neurological disorders.

